# Conclusions of the African Regional GIS Summit (2019): using geographic information systems for public health decision-making

**DOI:** 10.1186/s12919-022-00233-y

**Published:** 2022-06-17

**Authors:** Godwin Ubong Akpan, Hani Farouk Mohammed, Kebba Touray, John Kipterer, Isah Mohammed Bello, Reuben Ngofa, Andrew Stein, Vince Seaman, Pascal Mkanda, Joseph Cabore

**Affiliations:** 1 World Health Organization Regional Office for Africa, Djoue, Brazzaville, Congo; 2 Bill & Melinda Gates Foundation, Seattle, Washington USA

**Keywords:** Africa, Decision-making, Geographic information systems, GIS, Polio, Public health, World Health Organization

## Abstract

The use of geographic information system (GIS) technologies to improve access to health is gaining momentum in Africa. This has become more pertinent with the increasing penetration of mobile-phone technology and internet use, and calls for innovative strategies to support implementation of the World Health Organization Sustainable Development Goals for universal health coverage on the continent. The huge potential benefits of GIS to advance health service delivery in Africa is, however, yet to be fully harnessed due to critical challenges such as proliferation of pilot projects, poor coordination, inadequate preparedness of the health workforce for GIS, lack of interoperability, and inadequate sustainable financing. To discuss these challenges and propose the way forward for rapid, cost-effective, and sustainable deployment of GIS, the African Regional GIS Summit was held in Brazzaville, Republic of the Congo, on 7–10 October 2019 under the umbrella of the AFRO GIS Centre.

## Background

The World Health Organization (WHO) Polio Eradication Programme (PEP) is leading the application of innovative technologies within national health systems and public health services in Africa to ensure successful and timely implementation of the Polio Endgame Strategy 2019–23 of the WHO Global Polio Eradication Initiative (GPEI). One type of technology—geographic information systems (GIS)—is receiving increasing recognition as a key factor in achieving national and regional public health goals based on the demonstrated evidence of its impact. Through providing real-time, location-specific information, and data collection and analysis, GIS eases disease surveillance, and allows timely, focused, and appropriate responses in times of health crises, facilitating disaster preparedness, targeted capacity building, and efficient use of health resources. Supported by field-data collection using mobile-based information-management platforms, GIS has been used successfully by PEP to support the implementation of regional and national agendas for disease and environmental surveillance, routine and supportive immunisation, information management, monitoring, and evaluation, and accountability tracking of teams and individuals at all levels.

The WHO attaches great value to innovative technologies and GIS in particular, demonstrated by the establishment of the AFRO GIS Centre by PEP in February 2017. The centre, which was inaugurated by the Who Regional Director for Africa in the presence of key partners and stakeholders, serves to support WHO Member States in adopting technologies to tackle health challenges. The centre ensures that national adoption of GIS is adequate and that the required capacity needed to cover relevant knowledge areas is established.

An increasing number of Member States in the WHO African Region are implementing GIS, recognizing its power in early detection of disease outbreaks and its potential in achieving public health objectives. In Uganda, for example, application of GIS in surveillance enabled effective detection and management of an outbreak of Ebola virus disease (EVD) in June 2020 before it became a crisis. The need to share experiences in GIS implementation from countries such as Uganda, as well as to satisfy the recommendation of the 2017–8 African Region GIS road map that a second phase of GIS implementation and rollout to Member States be undertaken over 2019–23, provided the basis for convening a forum of the African Region’s GIS public health stakeholders in Brazzaville, Republic of the Congo, on 7–10 October 2019. The meeting was also intended as an opportunity to jointly plan GIS-related activities for the Polio Endgame Strategy 2019–23, which required reviewing the progress and challenges of GIS implementation in Member States, and determining how innovative ideas, potential collaborations, and available resources would be brought to bear on the wide-scale and successful use of GIS.

### Objectives

The general objectives of the African Regional GIS Summit were to review and discuss progress in the implementation of GIS and other innovative technologies, and lay out a national road map for Member States, WHO, and partners for 2020–1. The specific objectives were:

- To review country GIS implementation status, challenges, lessons learnt, and plans;
- To share the WHO GIS agenda, and progress in GIS implementation at global, regional, and country levels;
- To discuss GIS activities, resources, and new trends among partners, WHO regions, and stakeholders; and
- To draft GIS activity recommendations for 2020–1 on regional and national levels, as well as for in-country support teams.

### Expected outcomes

Several outcomes were expected:

- WHO Member States and partners would be briefed on progress in the implementation of the WHO Regional Office for Africa agenda for GIS;
- Awareness would be raised among Member States about potential benefits of GIS and new trends in GIS implementation;
- Lessons learnt and gaps would be recognized and documented, eg, infrastructure, applications, baseline maps and imagery, policies, and human and financial resources;
- The 2020–1 African Region GIS road map would be discussed and drafted for all stakeholders at all levels; and
- Potential collaboration and resource mobilisation opportunities would be recognised and planned.

### Participation

The meeting brought together representatives from all 47 Member States in the WHO African Region to learn how they could use GIS technology for public health decisions. Other participants included WHO staff; partners such as the United Nations Children’s Fund, Bill & Melinda Gates Foundation (BMGF), and Centers for Disease Control and Prevention; key stakeholders; GIS country focal persons; and private sector data analytics and GIS actors. The meeting modalities involved plenary presentations from WHO, the countries, and partners; technical breakout sessions to expose public health technicians to the latest developments in health-data collection and processing, and to engage with experts on the technologies from WHO, as well as partners and third-party representatives; and working groups comprising mostly policy makers who were entrusted with the duty of generating recommendations for developing the 2020–1 African Region GIS road map.

### Member States’ national capability for geographic information systems survey

To lay the groundwork for strategising and planning for the 2020–1 GIS agenda, the AFRO GIS Centre conducted a survey of Member States’ national capability for GIS from February to July 2019, in which the countries were asked to evaluate their GIS capacity in terms of three GIS pillars:

- Foundation and national capacity: workforce and capacity building factors, baseline data resources and standards, and infrastructure;
- Applications and information use: availability of documentation and research, software applications and services, methods, processes, and standard operating procedures; and
- Strategy, investment, and resources: legislation and policies, leadership and governance, and communication.

The three pillars had been defined with the goal of ensuring that the elements considered essential for sustained establishment of enterprise GIS capacity in the Africa Region were captured. All 47 Member States in the WHO African Region responded to the survey. The survey outcomes were used as the basis for grouping Member States with regard to GIS capacity—ie, belonging to the early-stage, midlevel (developed and building up), or advanced (scaling up and mainstreaming) category—and for drawing up recommendations from the GIS meeting for Member State, WHO, and partner action.

## Presentation summaries

### Harnessing innovative technologies and geographic information system applications for polio eradication and other immunisation/public health activities

#### Pascal Mkanda

##### Polio Eradication Programme Coordinator, World Health Organization Regional Office for Africa, Brazzaville, Congo

The AFRO GIS was established in February 2017 with the aim of supporting WHO Member States to adopt the latest information-capturing and analytic technologies to tackle health-sector challenges. Harnessing the power of such technologies, the centre facilitates data collection, analysis, and access to provide efficient, cost-effective, and timely delivery of public-health interventions. The centre currently uses five core applications for its field interventions: Autovisual Acute Flaccid Paralysis (AFP) Detection and Reporting (AVADAR), a community-based surveillance reporting tool; integrated supportive supervision (ISS); electronic surveillance (eSurv); digital-elevation models (DEMs); and the Vaccination Tracking System (VTS).

The AVADAR application is intended to improve performance of surveillance of polio and other diseases in areas where infrastructure problems exist and community reporting is used. A mobile-phone, short-message service (SMS)–based application, AVADAR allows disease reporting by health workers and community informants to a central server. Currently, 10 countries have adopted AVADAR, and 6611 community informants and 1350 health workers are involved. In 2018, 909 AFP cases were reported through AVADAR.

The ISS application combines active surveillance and immunisation assessments in one electronic tool. Over 160,000 supportive supervisory visits were carried out during 2018 in 42 countries. In 2019, Seychelles and Mauritius became the 43rd and 44th ISS-reporting countries, respectively.

The eSurv application enables active surveillance and monitoring of disease outbreaks by use of smartphones and with the help of an electronic checklist. It is focused on reporting surveillance from various sites including health facilities and traditional reporting sites, eg, patent medicine vendors, traditional healers and birth attendants, faith-based healers, and community informants. It also ensures that information reporting is in real time and it shows gaps in surveillance in real time.

A DEM is a 3-dimensional representation of a terrain or surface with elevation data. These models offer the benefits of clearly defining an environmental surveillance catchment area, providing a better estimate of a drainage population, and helping avoid interference in a drainage area of environmental surveillance sites.

The VTS helps determine where a vaccination has or has not been done. The VTS module is encapsulated in mobile phones carried around by vaccinators. It captures Global Positioning System (GPS) tracks as vaccinators go about their vaccination activities. The tracks are assessed against satellite maps of an area to check for coverage and gaps on a daily basis. This makes it possible for vaccinators to return and vaccinate those children missed the previous day.

The GIS technologic innovations can contribute to the achievement of universal health coverage through: enabling the involvement of community informants where health services are nonexistent; allowing monitoring of the accessibility and reach of health interventions, health-facility functionality, and quality of supportive supervision; and facilitating coverage surveys, estimation of populations at risk of a disease, and definition of the determinants of mortality.

### National assessment of geographic information system adoption in Africa: results and findings

#### AFRO GIS Centre Team, Brazzaville, Congo

##### Survey method and data collection process

The PEP undertook the first WHO African Region GIS assessment to understand the readiness and use of GIS in Member States. The survey aimed to determine the opportunities, successes, and challenges in each country to develop recommendations on how to enhance the adoption of GIS and innovative technologies, and to develop a national road-map toolkit that would guide countries towards establishing enterprise GIS and routinising GIS services within the public-health sector. The survey ran from February 2019 and all 47 Member States submitted their profiles before the end of September 2019.

Only one submission was required per country. The survey administration was designed to be straightforward and streamlined, particularly because it was using a platform that supports the use of both personal computers and mobile devices. Online completion was performed by the survey coordinator on receiving input from country informants at the survey meeting, and any extra data elements required were added after additional research and use of secondary data sets.

The national survey coordinator, who was usually the national GIS focal point, was requested to invite three–five experts to form a national experts group to work on the survey, reaching a consensus on each answer. The coordinator then submitted the completed survey online as a final record of the national response. The survey instructions included a list of key stakeholders who would be part of the survey working group, who were drawn from ministries of health, national laboratories, ministries of telecommunication, and information and communications technology (ICT), national mapping agencies and statistics bureaus, and nongovernmental organisations engaged in GIS.

The survey comprised 92 questions organised into 7 thematic areas: (1) GIS foundations, (2) baseline data and standards, (3) capacity building, (4) services and applications, (5) evaluation, (6) challenges in GIS implementation, and (7) lessons learnt. The questions covered national strategies, policies, rules and regulations, and mapping services and agencies, ministry of health GIS services, capacity, printing facilities, and baseline data sets and standards.

##### Survey findings

The survey revealed that 74% of 47 Member States had established a national mapping agency that could support the updating of national geographic profiles. Administrative boundaries were up to date for 64% of countries at the provincial level, 51% at the district level, and 28% at the subdistrict level. Only 45% of countries had an updated master list of population settlements.

The health geoprofile of the countries showed that 43% had an updated master list of health facilities, 26% had updated boundaries for health districts, 28% had an updated master list of georeferenced schools, and 43% had updated road maps.

The respective ministries of health in 28% of countries had a proper allocated working space for GIS services, 21% had GIS printing hardware and facilities, and 9% had dedicated servers for GIS applications and Web-based services.

Only 45% of Member States provided GIS training for their staff during the previous 2 years and it targeted mainly national ministry of health staff. Only 11% of the activities were meant for provincial staff and almost none for district staff. Electronic and distance learning methods were not used for GIS training activities, whereas 40% of countries used mainly instructor-led methods and 15% used on-the-job training. The survey found some capacity gaps in the skills to support GIS activities in the health ministries. There was a lack of availability in GIS specialists in 85% of countries, project management specialists in 81%, remote sensing experts in 85%, and GIS programmers in 89%. Training in GIS was not usually provided locally; ~ 90% of training required travelling, 29% of which was domestic, 24% international, and 29% both.

Open-source and licensed software were used collaboratively in the WHO African Region, although several ministry of health staff were using outdated and obsolete versions of GIS tools such as ArcView 3.x (19%) and HealthMap (45%), both of which are no longer supported by their developers.

In all, 51% of countries had established GIS services and applications, among which 73% were operational, 55% did not have any GIS staff at the ministry of health, and 43% had 1–10 GIS staff.

The focus for the generation of maps and GIS information products was mainly internal ministry of health departments for 83% of countries and international organisations for 66%. Ministry of health directors as a target for the products came next with 57% of countries, followed by ministers of health at 53%. In general, there was low support in the form of rules and regulations for the use and dissemination of geodata, and only 28% of countries had such tools.

Of the 47 countries, 63% had national ICT strategies, among which 32% covered GIS.

##### Challenges in geographic information system implementation in the WHO African Region

The Member States identified 14 key challenges, which they also ranked. The lack of field equipment was the top challenge, scoring 78%, and was even more important than the lack of funding to support GIS services at the respective ministry of health (75%). A clustering analysis based on the challenges identified by the countries and using the k-means technique classified the 47 countries into three distinct groups:

- Cluster 1: Angola, Benin, Cape Verde, Chad, Democratic Republic of the Congo, Equatorial Guinea, Eritrea, São Tomé and Príncipe, South Africa, and Uganda;
- Cluster 2: Algeria, Gabon, Ghana, Lesotho, Mauritius, Mozambique, Namibia, Nigeria, Rwanda, Seychelles, Tanzania, and Zambia;
- Cluster 3: Botswana, Burkina Faso, Burundi, Cameroon, Central African Republic, Comoros, Ethiopia, Cote d’Ivoire, Eswatini, the Gambia, Guinea, Guinea Bissau, Kenya, Liberia, Madagascar, Malawi, Mali, Mauritania, Niger, Republic of the Congo, Senegal, Sierra Leone, South Sudan, Togo, and Zimbabwe.

### Innovative strategies for polio eradication: global geographic information system experience

#### Ravi Santhana^1^ and John Kipterer^2^

^*1*^ *World Health Organization, Geneva, Switzerland*

^*2*^ *World Health Organization, Regional Office for Africa, Brazzaville, Congo*

Boundaries change often as a consequence of policies and population-census factors, and for administrative purposes, eg, to form new or eliminate existing administrative units, or rename administrative units. Tracking historical boundaries and storing historical boundary data are important for both posterity and trend analysis, and WHO has made it a responsibility to maintain data on old and changing geographies using the concept of time-associated polygons. Any global level boundary change starting from 2000 is maintained in shapefiles in the WHO global geodatabase.

The WHO PEP boundary project compiles in a central place ~ 50,000 historical boundaries, as well as subnational health boundaries, stored in a manner to allow trend analysis, eg, by maintaining in shapefiles polygons of previous and current boundaries, and associated populations. These boundaries cover the six WHO regions and are updated regularly. The PEP boundary project is also working to generate fine, detailed coastline and country boundaries for WHO purposes, mostly to allow integration of data coming from the field, which have exact location coordinates, and to accommodate generation of interactive maps. It is possible also to overlay the boundary data with the population data from the United Nations Development Programme, Landscan™ (Oak Ridge National Laboratory, Oak Ridge, Tennessee, USA), WorldPop (Southampton, UK), and the countries to generate data sets segregated by age and gender. The use of drones for medical deliveries, which relies on geocoordinates for location definition, makes GIS-based boundaries essential, as does the need to generate precise, nonconventional data sets such as those used by the United Nations in classification of location security. Nonconventional data sets are useful for programmes as they permit analysis of security incidents and determination of their likely impact on the programme.

The PEP boundary project is providing a place where people can obtain a population data set that brings together data from various sources and stores them in a harmonised manner.

### Technologic innovations to support the effort toward certification of poliomyelitis eradication in the WHO African Region

#### Koffi Kouadio

##### Regional Polio Certification Officer, World Health Organization Regional Office for Africa, Brazzaville, Congo

The WHO African Region has been tracking and reporting wild poliovirus (WPV) since 1997 and has seen cases drop drastically, going from the highest level of 1192 in 2006 to 4 in 2016, the last year the disease was reported in the region. For 32 of the 47 Member States, the last case of WPV was reported > 10 years ago. Since the last case was reported on 21 August 2016, no sample has been tested positive for WPV. This is, however, not the situation with circulating vaccine-derived poliovirus 2 (cVDPV2), where the number of countries reporting the disease grew from 1 in 2016 to 9 in 2019. Several measures have been put in place to tackle this cVDPV2: (1) a GPEI Rapid Response Team (RRT) was established in September 2019 in the region; (2) all Member States have revised their polio-specific preparedness and response plans within the last 3 years; (3) campaigns and surveillance-strengthening initiatives have been instituted to respond to cVDPV2 outbreaks; (4) nonpolio AFP rates and stool adequacy for 1997–2019 were met, as were the 2016–9 AFP national level surveillance performance rates; (5) environmental surveillance was started in Nigeria in 2011 and in 36 other countries in 2019; and (6) immunisation programmes are conducted in all the countries, although the global shortage of inactivated polio vaccine affected the programmes’ performance.

Activities to strengthen AFP surveillance and immunisation include: (1) the Brazzaville initiative for strengthening surveillance in high-risk countries; (2) the Lake Chad Basin task team initiative in Cameroon, Central African Republic, Chad, Niger, and Nigeria, and areas with insecurity concerns; (3) military involvement in surveillance and vaccination activities in insecure areas, such as northern Nigeria; (4) collaboration with nongovernmental organisations and humanitarian agencies in the Central African Republic, Chad, Mali, and South Sudan; (5) expansion of community-based surveillance by increasing local informants in insecure areas; (6) expansion of environmental surveillance; and (7) institutionalisation of the accountability framework to improve staff and programme performance at all levels. Technologic innovations for strengthening surveillance and immunisation activities include: (1) GIS technologies for case tracking and provision of real-time evidence in surveillance activities; (2) AVADAR use for reporting by communities focusing on high-risk areas; (3) ISS application to strengthen surveillance and routine activities; (4) geomapping to define areas of concern and improve microplans for better campaign quality; and (5) eSurv use for real-time information sharing on field activities.

Key challenges facing polio eradication include: (1) ongoing cVDPV2 outbreaks; (2) reaching children in security-compromised and inaccessible areas; (3) large movements of internally displaced people, refugees, nomads, and pastoralists; (4) surveillance gaps, particularly where districts do not meet indicators or are silent and where subnational surveillance gaps exist, bringing with them the risk of missing transmission events; (5) population immunity gaps; and (6) lack of compliance by countries to set deadlines for submission of risk-assessment reports.

Priorities for 2019–20 include: (1) interrupting poliovirus transmission in outbreak countries; (2) scaling up the use of GIS technologies to strengthen surveillance and routine immunisation; (3) supporting countries to achieve containment in line with guidance for potentially infectious material; (4) promoting regular use of risk assessment and implementing mitigation plans; (5) supporting the preparation for certification of the four remaining countries whose documentation is pending acceptance by the Africa Regional Certification Commission for Poliomyelitis Eradication; and (6) monitoring country progress towards regional certification.

### Mapping of the nomadic population: Lake Chad Basin experience

#### Ajiri Atagbaza,^1^ Isaias Fernandes^2^ and Tess Palmer^3^

^*1*^ *GIS Officer, World Health Organization Regional Office for Africa, Brazzaville, Congo*

^*2*^ *United Nations Volunteer, World Health Organization Regional Office for Africa, Brazzaville, Congo*

^*3*^ *Geospatial Research, Analysis, and Services Program, Centers for Disease Control and Prevention, Atlanta, Georgia, USA*

To tackle polio outbreaks in Nigeria, the Lake Chad Basin task team centered its efforts during the first four phases of its response to strengthening the capacity of the Lake Chad Basin countries, increasing immunity of the populations against polio, and strengthening AFP surveillance by making it more sensitive to the detection of poliovirus. Some high-risk populations such as nomadic groups who move freely about the region were, however, not reached by these efforts. It became necessary, therefore, to track them and make sure they were part of the planning activities. At present, 13 nomadic groups have been identified.

The fifth phase of the project, running February–August 2019, included nomadic groups that had been missed out previously, with no documented evidence of their movements. A workshop with representatives from Cameroon, Chad, and Nigeria was held to come up with ideas on how nomads could be identified. A data kit was developed incorporating an online survey form and operational manual, which were test run during the workshop. Discussions were held with nomad experts to identify nomadic groups.

The population of nomads in the area was ~ 35,000, but that number may have since increased. The proportion of actual nomads that were reached was difficult to determine without a baseline. Estimates showed that 3–5% of WPV cases in Nigeria were in nomads and this would likely increase to 10% if those in close contact with these cases were included. Nomads play an important role in polio transmission and some cVDPV2 cases have been associated with their movements.

Achievements of this project include: (1) identification of actual nomadic groups, who were each given unique codes; (2) development of a regional mobile data-collection to capture the locations of nomadic camps, as well as other variables on the time of inhabitation, the group at the camp, previous and future camp locations, camp population, and AFP case surveillance; (3) data collection and hosting on the WHO African Region server; (4) training of data collectors in the Lake Chad Basin countries, ie, Cameroon, Central African Republic, Chad, Niger, and Nigeria; (5) enabling the development of mapping and visualisation products to show movements of specific nomadic groups, and support planning and decision-making at different administrative levels.

Challenges of this project included: (1) time constraints that prevented coverage of a complete cycle of any nomadic group’s migration; (2) ddifficulties in generating correct unique group codes; (3) lack of data-quality ambassadors at country and district levels; (4) insufficient training of data collectors (dedicated data collectors with nomad data collection as part of their terms of references are required); and (5) closure of the Lake Chad project due to insurgency, which may affect the gains made against polio in the region.

Next steps include: (1) assessing all partner views and ensuring all data being pulled in and merged are working correctly so that they are immediately accessible for export via the Ona platform (Ona Kenya, Nairobi, Kenya); (2) identifying a data-quality ambassadors at the country level to assess the data submitted and fix issues as they arise; (3) identifying a data manager at the WHO Regional Office to communicate with country-level data-quality ambassadors, fix group code issues, and make changes to the ODK form as needed; (4) working with the Centers for Disease Control and Prevention to develop action plans for creating a tool kit for implementing this project in other countries in the region, as well as a standardised training package; (5) providing each country with basic maps of all health administrative levels to use in the field, which will assist data collectors in determining their location and reduce unknown responses; (6) development of a plan by the Regional Office for cross-border communication and tracking of groups that cross international borders; and (7) creation of a monthly sitrep template for reporting on nomad data or configuration of an automatically updating dashboard.

### Outsmart outbreaks with innovative digital health interventions: African Region field-data collection experience

#### Godwin Akpan

##### World Health Organization Regional Office for Africa, Brazzaville, Congo

It is in the interest of the WHO PEP that relentless focus be directed at the small areas where the virus may still present, children are being consistently missed, immunity levels are low, or surveillance is weak. The areas of concern at the moment are Burkina Faso, Central African Republic, the Horn of Africa region, the Lake Chad region, Mali, Niger, and South Sudan. The PEP approach involves building synergistic relationships for the realisation of its goals. In regard to the use of GIS innovations where the interest is in adding geographic information to decision-making, information generated from the various tools is amalgamated to generate a 360° view of the situation. The first step involves community-level detection of an AFP case, which is reported in real time through an AVADAR SMS or email message to the next level, which could be a health facility, and then to the district, provincial, national, regional, and global levels. What is important here is the geographic information, without which establishing the location of the child, facility, or informant would be difficult, and with which a lot more can be accomplished than was previously possible, eg, an intervention can be planned and implemented faster. Only 10 countries in the WHO African Region are using AVADAR, which is unfortunate considering that its use is known to increase detection of AFP by as much as 40% and community reporting by up to 98%.

The high mobile-phone penetration in the African Region is another important piece of the puzzle. It has contributed to the improvement of data quality and helped reduce the cost of collecting data. The use of eSurv also has helped in monitoring AFP reporting, particularly for traditional sites where rural people or poor urban dwellers seek medical help, keeping everyone informed via SMS or email.

Through use of ISS, which is available in 44 countries, the quality of surveillance and routine immunisation practices can be checked by harnessing data from systematic visits to priority sites for assessment, evaluation, and on-the-job training for health workers. When all the information from AVADAR, eSurv, and ISS is compiled for decision-making and deeper analytics using maps, a gradual closing of the gaps in surveillance and routine immunisation occurs, which is now happening in the Sahel region countries of Burkina Faso, Mali, and Niger, and Lake Chad region countries of Cameroon, Chad, Niger, and Nigeria. Information synthesis using GIS helps narrow the focus in small areas with surveillance gaps and predicts health-system resilience in areas that have been assessed.

Immediate challenges include: (1) the limited availability of telecommunication networks in some locations and their cost; (2) the innovations’ scope limitations, eg, AVADAR is limited in use due to the cost of its implementation; (3) lack of validated comprehensive lists of health facilities for the countries in ISS and eSurv to support health-facility monitoring; (4) reluctance to implement the innovations, which generally provide real-time data in an objective manner and are useful for staff accountability, an attribute that staff may not like; (5) not all countries are on board with the technologies (missing countries include Algeria, Cape Verde, and Comoros; and (6) levels of implementation and utilization differ across countries due to differences in context.

The way forward will involve: (1) scaling up the use of GIS-based mobile digital interventions such as AVADAR, ISS, eSurv, and others beyond 2019; (2) updating the lists of health facilities in countries, and providing unique IDs in ISS and eSurv for each facility; (3) starting the process of validation visits to monitor the effects of supportive supervision and ensuring the high quality of the data sent to the server; (4) continuing to use the innovations to streamline electronic surveillance site validation and selection from 2020 to 2023, as outlined by the GPEI; and (5) continuing capacity building for government counterparts on GIS, digital-health leveraging, and data-visualisation applications to cover the remaining countries in the African Region.

### Rapid response team: a new initiative to support outbreak response

#### Modjirom Ndoutabe

##### African Region Rapid Response Team, World Health Organization Regional Office for Africa, Brazzaville, Congo

Since 2017, African countries have been confronted with a heavy burden of cVDVP2, and even severe and long-lived outbreaks have occurred in some countries. In response, the GPEI established a full-time RRT in Africa that is intended to serve as the first boots on the ground during an epidemic. The team is usually deployed for 6–8 weeks following an outbreak and works closely with all partners involved in the country.

The RRT’s responsibilities include: (1) dealing with all affairs related to outbreak response, such as provision of technical oversight, strategy development, activity definition and coordination, partnership building, and partner support and liaison (note: all activities in the countries must be undertaken with the approval and involvement of the relevant government bodies and other stakeholders); (2) enhancing surveillance, and using technologic innovations in both surveillance and response; (3) providing technical support to the countries to enhance their AFP surveillance; (4) building the capacity of the countries in the use of eSurv, GIS, ISS, and other innovations using smart phones; (5) providing technical support to countries in finalisation of their polio certification documents, and helping those countries address gaps and challenges before presentation of their documentation to the Africa Regional Certification Commission for Poliomyelitis Eradication; and (6) supporting polio-free countries in meeting surveillance standards and immunisation indicators required to maintain their polio-free status.

Deployment of RRT could be deterred by delays occasioned by visa requirements for some countries, lack of flights, or the failure of a country to receive the team members. In outbreak situations, RRT should be deployed within 24–72 hours, depending on the circumstances related to the outbreak.

### Geospatial approaches and applications during the response to the Ebola virus disease outbreak in the Democratic Republic of the Congo as an example of geographic information system activities within the WHO Health Emergencies Programme

#### Roland Ngom

##### Technical Officer–GIS, World Health Organization Regional Office for Africa, Brazzaville, Congo

In its work to fulfil its mandate of minimising the health consequences of outbreaks and emergencies, the WHO Health Emergencies Programme (WHE) African Region’s section ensures that GIS tools are a functional component of outbreak management, and are integral features in outbreak surveillance and detection, preparedness, intervention, and recovery. The WHE works with the countries to plan for various GIS solutions to prepare for outbreaks and to tackle outbreaks when they occur, ensuring that capacity on GIS basics and tools is built before and during outbreaks.

The WHE was instrumental in the efficient handling of the EVD outbreak in the Democratic Republic of Congo that started in 2018. Through providing history maps of EVD epidemics in the country, heat maps of security incidents, and EVD maps for reporting, WHE facilitated targeting of planning for the epidemic and resource allocation. A GIS human hub was created to complement the human resources in the country and harmonise GIS tasks. Before GIS tools were integrated as a key component, data collection and workflow were segmented.

The use of GIS tools also allowed: (1) evaluation of existing geolocated data and geographic products; (2) identification of geolocated information and geographic products; (3) avoidance of effort duplication and sharing of existing products; (4) completion of missing geographies and correction of wrong ones; (5) on-site training on data collection and basic mapping with the field and ministry staff, nurses, doctors, and local communities; (6) setting up an online central repository for hosting and publicly sharing filtered geographic and nongeographic data; (7) a geospatial, data-driven response approach permitting daily epidemiologic situation mapping at the finest spatial health geography level, showing healthcare-seeking movements and identifying health facilities near clusters of cases for response-activity prioritisation; and (8) online interactive dashboards and applications for various aspects of the response, showing the situation on the ground, human resource deployment, vaccination coverage, and epidemiologic surveillance.

Lessons learnt include: (1) before and during an outbreak, preparedness is key; (2) GIS tools should be fully integrated in existing tools and processes for data collection and storage, and information visualisation; and (3) GIS tools are important in all outbreak-response aspects, not just epidemiologic surveillance.

The way forward will involve: (1) harmonising deployed tools and ensuring clear definition of roles; (2) introducing more efficient data-management processes; and (3) standardising data collection, storage, and sharing procedures.

### Why the immunisation community needs geographic information systems and technology: the case of the African Region

#### Alain Poy

##### Technical Officer–Immunization Data, World Health Organization Regional Office for Africa, Brazzaville, Congo

Africa’s economic burden from the vaccine-preventable diseases (VPDs) pneumococcal diseases, rotavirus, measles, and rubella stands at US$ 13 billion. Previously, it was difficult to implement immunisation plans for VPDs because data were not available or were of inadequate quality. Progress has now been made with the integration of immunisation and VPD surveillance data within District Health Information Software 2 (DHIS2). With this one information system, it is easy to identify children who are missed out in immunisation, avoiding the usual problem where the same children continue to be immunised.

The WHO considers GIS applications as crucial tools in the efforts to meet immunisation goals for VPDs and DHIS2 configuration packages are the preferred choice in these efforts. The DHIS2 health applications are based on international standards. They facilitate the consolidation of health-facility data from multiple health programmes to allow use of a comprehensive, integrated approach in immunisation programmes for VPDs. This allows for a crosscutting overview of the services’ performance for planners and managers for better decision-making on improving health services. Eighteen countries in Africa are using DHIS2 applications currently.

Three product packages in the applications are particularly useful in VPD immunisation programmes. The DHIS2 dashboard allows core analysis of immunisation doses, and coverage data for vaccines, dropout rates, supply, and cold-chain factors. The immunisation analysis application is concerned with tables of vaccine doses and immunisation coverage, the immunisation monitoring chart, and charts and tables of categories based on diphtheria-pertussis-tetanus-1 coverage and dropout rates. The WHO data-quality tool conducts data analyses on the dimensions of data timeliness and completeness, internal and external consistency, outliers, and trends.

Geographic information systems can help improve immunisation by: (1) tracking immunisation outreach sessions; (2) tracking population movement for routine immunisation, going beyond supplementary immunisation activities (SIAs); (3) ensuring data quality by verifying the effectiveness of service delivery for routine immunisation; (4) reinforcing the use of satellite imagery for routine immunisation microplanning, operational denominator determination, and identification of hard-to-reach or unreached children; and (5) facilitating new technology for vaccine delivery.

Geographic information systems can contribute toward achieving universal health coverage in the WHO African Region by: (1) using community informants where health services are nonexistent; (2) monitoring accessibility and reach of health interventions; (3) monitoring health-facility functionality; (4) undertaking coverage surveys to identify populations and areas not reached by an intervention; (5) defining determinants of mortality to increase coverage of interventions; (6) monitoring the quality of supportive supervision jointly undertaken by government and partner staff; and (7) estimating populations at risk of a disease.

The way forward will involve: (1) including a GIS strategy within the context of Member States’ overall health-information system strategies; (2) as a legacy, positioning the GIS structure as a crosscutting component within the health-information or -system strengthening structure; (3) ensuring resource mobilisation for GIS sustainability beyond GPEI; and (4) reinforcing national capacity for robust and integrated health-information systems.

### Global geographic information system resources, licenses, databases, online platforms, and satellite imagery

#### Ravi Santhana,^1^ John Kipterer,^2^ Frank Salet^3^ and Travis Butcher^4^

^*1*^ *World Health Organization, Geneva, Switzerland*

^*2*^ *World Health Organization, Regional Office for Africa, Brazzaville, Congo*

^*3*^ *Bill & Melinda Gates Foundation, Seattle, Washington, USA*

^*4*^ *Esri, Redlands, California, USA*

A review of GIS operations at WHO found that sustaining and running a GIS infrastructure required governance, investment, geodata, capacity, a platform, strategy, and delivery. The WHO headquarters GIS infrastructure encompasses, however, only geodata, a platform, and GIS capacity. This means that WHO Member States may also have weak GIS infrastructures. Factors such as data ethics and legal aspects, data interoperability, and data-management practices are also important. The data input that Member States provide to the WHO GIS infrastructure are the reference data set, working data set, and information products.

The reference data set has three components: (1) The utility data set is the population data set, and includes district and province names, ie, geography, but not the associated spatial data. (2) A time component is added to the population data, as well as gender and age attributes; however, there should also be an association between time and geography. (3) The geodata is a shapefile with two components: geography, ie, the names of level-1 and -2 administration structures, and geometry, ie, the shape associated with the geography. The shapefile allows visualisation of the location and the time factor associated with it.

The working data set, which is updated daily (except for AFP data, which are submitted weekly), has time and geography associated with it. For example, laboratory and AFP samples have collection location and tracking information. Data from countries with polio outbreaks, and from high-risk countries where SIA data are collected or polio campaigns are run and monitored, include time, geography, and population aspects.

Information products are outputs generated from the data sets provided by the countries. The WHO headquarters GIS facility generates indicators (eg, for stool adequacy and immunity profiles), as well as analytics such as maps and charts. It also conducts strategic interventions such as mapping of nomadic populations and sentinel sites.

Challenges occur when the utility database geography and shapefiles do not match. The utility database is updated once a year, while districts and provinces captured in the shapefiles are formed during the year, which is the basis for AFP surveillance and case reporting. This mismatching affects analytics on polio. Another issue is inconsistencies in the data collected. For example, AFP case data collection requires the utility database to support the geography and there will be problems if this does not occur. Such problems will not arise if the utility database has accurate and complete information, and its use is enforced.

Further, problems occur when SIA and surveillance data are not talking. If SIA teams use their own geography and collect their own district names, problems will occur when indicators need to be generated. Different spellings of district names will also cause a similar problem.

### The Africa GeoPortal

#### Travis Butcher

##### Esri, Redlands, California, USA

The Africa GeoPortal is a central location providing data, tools, and support for analysing, visualising, and discovering different aspects of Africa. It is available free for those on the continent or working to support work on the continent. Data on the portal is drawn from Esri (Redlands, California, USA), governments and development bodies, users, and commercial enterprises. It uses ArcGIS Online, which enables finding, creation, and storage of data on the Web, and making it available for access to others. The portal facilitates collaboration, improves efficiency, and provides access to various geospatial tools, which can be used to visualise and enrich data. More information is available from the GeoPortal Website (https://www.geoportal.org).

### Deep learning applied to remote-sensing and geospatial data, and technologies based on experience from the polio programme in Nigeria

#### Stéphane Vouillamoz

##### Novel-t, Geneva, Switzerland

Collection of geospatial data makes it easy to manipulate and analyse data for informed decision-making targeted to specific locations, and to guide people to those locations. In supervisory surveillance, use of geospatial data allows verification that work was performed, how it was done, and if it met the set requirements. A good GIS map is required to get maximal benefit from geospatial data. Such a map should contain boundaries, settlements, population estimates, points of interest, and roads. Maps can help determine the numbers and locations of health facilities and people living within 5 km of a health facility, which can allow determination of resource requirements. With geospatial data, microplans can be generated and when such data are centralised, cross-interventions and time-trend analyses can be generated, guiding field staff.

Analysts’ observations from geospatial data have been used to train deep-learning models. Such models can effectively automate image-classification tasks to make predictions. Deep-learning models generate predictions as soon as new imagery is available to them. Their use in predictions reduces the workload of analysts. In Kano State, Nigeria, use of deep-learning models in a VTS led to a reduction in settlements chronically missed in polio vaccination—from 38 in January 2014 to 19 in March—following introduction of a “Hamlet Buster” deep-learning model in February.

### Use of geographic information systems in the Neglected Tropical Disease Programme

#### Noémie Y. Nikiema Nidjergou

##### Neglected Tropical Disease Programme, World Health Organization Regional Office for Africa, Brazzaville, Congo

The Neglected Tropical Disease Programme (NTD) life cycle comprises three stages, all of which benefit from the use of GIS, which enhances data collection, analysis, and access. During disease mapping and plan development, GIS supports the selection of survey sites and delineation of intervention zones. In programme implementation, GIS is used for identifying treatment gaps, and during disease elimination and surveillance, it facilitates visualisation of areas no longer requiring treatment interventions. To push the benefits of GIS tools close to where they are needed to accelerate the elimination of the five most prevalent neglected tropical diseases (lymphatic filariasis, onchocerciasis, soil-transmitted helminths, schistosomiasis, and trachoma), and in an effort to mobilise political, technical, and financial resources, the WHO Regional Office, Member States, and NTD partners established the Expanded Special Project for Elimination of Neglected Tropical Diseases (ESPEN) in 2016. The ESPEN aims to equip all stakeholders with the evidence they need to successfully tackle each disease, mobilise resources more efficiently, and target interventions appropriately.

Established in the spirit of public-private partnerships, ESPEN enables coordination among ministries of health and their stakeholders through the ESPEN Portal, which is an electronic platform designed to enable them to share and exchange subnational programme data in support of the NTD control and elimination goals. Using maps, site- and district-level data can be aggregated by disease and country. These maps, along with the downloadable underlying data, are tools to aid health officials and their partners in boosting and developing NTD interventions and strategies to reach the targeted communities. Interactive co-endemicity and mass drug administration regimen maps provide a rapid picture of the current populations requiring treatment. Further, through a free mobile data collection tool, the portal supports national programmes to submit survey protocols for refinement.

The ESPEN provides technical and operational support to endemic countries in their efforts to control and eliminate targeted preventive chemotherapy–neglected tropical diseases by: ensuring 100% geographic coverage for interventions; scaling down, stopping treatment, and acquiring WHO validation as soon as elimination is reached; strengthening information systems by allowing access to high-quality data; improving utilization of donated medicines to reach those who need them; and fostering partnerships, advocacy, and resource mobilisation.

### Use cases of geographic information systems and innovations in the African Region: scenarios beyond polio interventions

#### Godwin Akpan,^1^ Daniel Oyaole^2^

^*1*^ *World Health Organization Regional Office for Africa, Brazzaville, Congo*

^*2*^ *World Health Organization Nigeria Country Office, Abuja, Nigeria*

Geographic information systems transform paper-based surveys and active-case searches on priority diseases into mobile-phone applications, which are then used to collect location coordinates. Collection of geographic data on points provides unlimited ability to generate and analyse data more accurately than ever before for more precise planning. From health-system performance and coverage data, gaps can be ascertained in real time and addressed expeditiously. Mostly, ODK is used. Although the WHO PEP was the gateway through which GIS applications came to the African Region, their adoption among programmes has been widespread.

Noma disease surveillance has benefited from GIS-based interventions, which facilitated the inclusion of a 1-page case-reporting form on mobile phones of all health workers using eSurv and ISS. This information, which was hitherto difficult to gather owing to the stigma associated with the disease, is collected with coordinates and is communicated in real time.

Usually in campaigns associated with adverse events, data are collected on paper. Many countries are now moving to electronic data collection for deeper insights into interventions and are using mobile phones for real-time data reporting. Uganda received GIS support in mapping the form of delivery of case-definition information to frontline workers in border locations next to the Democratic Republic of the Congo during its EVD preparedness-response activities. For Nigeria, GIS has been used to map the establishment of facilities offering directly observed short-course treatment for tuberculosis and the quality of service, as well as in cold-chain assessments. Other countries using GIS include: Liberia, where GIS was used in measles-coverage surveys to identify areas with children not vaccinated in previous campaigns; the Central African Republic, where it was used in active-case search for priority diseases; South Sudan, in which it facilitated sampling tracking for MenAfriVac coverage; Zambia, where it was used for cold-chain assessment with ISS; Ethiopia, where it was used for data-gathering information on acute watery diarrhoea in Jijiga in the Somali region; and Lake Chad Basin countries, where it is used for tracking international and internal nomadic movements for improved targeted vaccination.

Nigeria’s public health sector has had wide engagement with the African Region’s GIS innovations, including in supportive supervision for routine immunisation, where dashboard heat maps indicate activities on the ground, going down to the ward level, if needed. For humanitarian and emergency support in the northeastern zone, mapping allows definition of interventions based on local circumstances. In the case of measles, as well as for all nonpolio SIAs, microplanning has been made easier with the use of GIS innovations, which allow predictive analysis and projections. Triangulation of laboratory-confirmed rubella case data with labour data has enabled prediction of related intervention time and resources. For yellow fever, the type of outbreak and spatial localisation data provided the information needed to determine the action to take.

Nigeria conducted a comprehensive cold-chain inventory assessment at all levels of the immunisation supply chain in 2018 with WHO innovations and GIS support. The goal was to accurately map and estimate the volume and functionality of cold-chain equipment within the supply chain for estimation of future requirements. Quality-assurance measures deployed for assessment included use of cold-chain equipment model and associated data from the WHO inventory-replacement planning tool; use of ODK for entry-data validation; mapping geolocation of equipment; and configuration of cold-chain equipment, and data cleaning, consolidation, and analysis, using the inventory replacement planning tool.

### Digitalisation of the health information system in Cameroon

#### Maurice Fezeu

##### Ministry of Public Health, Yaoundé, Cameroon

Four years ago, no data were available at the national level from any hospital in Cameroon and neither was a data-management system in place. Data collection was structure specific, with 13 structures collecting parallel data in 2094 facilities. A decision to integrate health data through digitalisation was made in 2015, beginning with the health map. The DHIS2 system was chosen for the digitalisation process. Numeration of health data started in December 2015, beginning with polio. Data were collected from all locations considered important, along with GPS coordinates of all health facilities, settlements, markets, schools, and churches, which allowed the creation of precise digital maps. People were trained in GIS software so that they could update their maps. Several other features were introduced: (1) the first national health map was produced based on the data collected; (2) data collection and management tools were harmonised and reduced from 28 to 1 form, and customised in DHIS2; (3) health denominators were harmonised to ensure that everyone was using the same ones; (4) PDFs of health maps were uploaded to the Website of the Ministry of Health for access by doctors; and (5) training in the use of the system was provided. Smart phones and tablets were distributed to staff to encourage them to use the system, which they had been reluctant to do at first. The DHIS2 system is used for all processes, including daily monitoring activities and even for distribution of bed nets. It is possible now to monitor cholera and represent on a map the points of main interest for those who need to know or even to know who went to a hospital with cholera symptoms.

The challenges associated with the digitalisation process include the lack of completeness of some reports, which is mostly associated with the failure to cater for English speakers, and the failure of policy-makers to participate in DHIS2 owing to their lack of training when that occurred for centre-level technical staff.

### Implementation of geographic information systems and innovative technologies in the Democratic Republic of the Congo: policies, applications, challenges, and lessons learnt

#### Jean Paul Makala,^1^ Robert Kuzanwa^2^ and Albert Mbule^2^

^*1*^ *World Health Organization Uganda Country Office, Kampala, Uganda*

^*2*^ *World Health Organization Democratic Republic of the Congo Country Office, Kinshasa, Democratic Republic of the Congo*

The Democratic Republic of the Congo is located in central Africa, straddling the equator and lying between the tropics. It has an area of 2,345,410 km^2^ and shares its 9165-km borders with nine countries. Owing to the lack of up-to-date population data, with the last census having been held in 1984, the health services use population data from microplanning and enumeration activities to plan and evaluate the performance of their interventions. The population was estimated at 104,234,095 in 2018. The country faces many communication difficulties owing to its large size, and populations in remote, insecure, or riparian areas do not receive health services in a consistent manner.

The GIS activities in the country fall under the Institut Géographique du Congo, a state service concerned with standardisation and management of policies relating to geographic systems. The GIS service provision confronts several challenges, including: GIS training is often incomplete and unsystematic; health-zone and health-area level maps are out of date; and the actors involved in the management of geographic information are not coordinated.

The need to run several campaigns against *cVDPV2* presented an opportunity to use new technologies, including GIS, in public health. These activities remain running and they include: drawing up microplans incorporating satellite-mapping data; digitalisation of villages in health zones using GIS tools; supervision of vaccination teams during *cVDPV2* responses using mobile phones; independent monitoring and lot quality-assurance sampling during cVDPV2 responses in 16 provinces using mobile phones (previously, health-zone teams had difficulty accessing such results owing to lack of access to a server); AVADAR coverage for the Rwashi and Tshamilemba health zones in Upper Katanga province, Mpokolo health zone in Kasaï-Oriental province, Tshikapa health zone in Kasaï province, Dilolo health zone in Lualaba province, and Walungu health zone in South Kivu province; implementation of ISS using mobile phones during January–September 2019; use of drones for vaccine distribution in health zones with difficult access; introduction of digital screens in national Expanded Programme on Immunization sites, COUP (the Emergency Operations Centre), and the Minister of Health’s cabinet offices for real-time surveillance of field-monitoring activities; and use of Go.Data software in electronic tracking of contacts in EVD surveillance and management in North Kivu.

Commitment to promote the use of digital technology in decision-making exists at the highest level of the government and has materialized into the implementation of the National Digital Plan. Partners such as BMGF have been mobilised for digital expansion in various fields, as have local telecommunication companies, and tools such as Facebook have also been put to use. In addition, the Ministry of Posts, Telecommunication and New Information and Communication Technologies was created, and a technical commission for the preparation of the second biometric census established.

Several applications are in use in the country, the choice of which is dependent on the partner supporting the service provided. They include Health Mapper, Epi Map, ArcGIS, ArcMap, QGIS, Quantum, and MapInfo (Precisely, Burlington, Massachusetts, USA).

The use of digital software in the public health sector is hampered by challenges associated with: lack of software standardisation; lack of use of GIS at intermediate and operational levels; lack of finalisation of the third administration layer, where of 26 provinces in the country, only Kinshasa, Kongo Central, Kwango, Kwilu, and Maindombe, and parts of Mongala and Haut-Lomami have had this done; issues relating to renewal of AVADAR phones and accessories; lack of training on and systematisation of the use of electronic forms with ISS and eSurv during field supervision; issues associated with creation of villages and their points of interest; inadequate financing of digitalisation activities for the third administration layer; lack of or poor access to the Internet for exploitation of data stored on the server; and acquisition of a systemised mechanism for obtaining contour updates and shapefiles pending.

The use of the new technologies during vaccination campaigns has facilitated monitoring of field supervisors and vaccinators.

### Niger experience on outbreak response

#### Moussa Haladou

##### World Health Organization Niger Country Office, Niamey, Niger

Niger was declared to have a polio virus break in 2015, but it continues to maintain tight surveillance over the disease because its neighbour Nigeria is at risk. The country’s geopolitical characteristics do not facilitate implementation of health services, with only 50% of the population having regular access to healthcare services. Some 15–20% of the population are nomads. Security issues in the Lake Chad Basin, and along the borders with Burkina Faso and Mali pose other threats.

On 21 September 2018, the presence of cVDPV2 was reported in Magaria and Tanout in the Zinder Region, and on 5 October, the epidemic was declared a public-health emergency of national and international importance. From the beginning of the outbreak to 22 July 2019, 25 cVDPV2 cases were reported in Magaria, Tanout, Dungass, and Bosso. These cases were found to be related to the outbreak in Borno State in Nigeria.

Vaccination campaigns were run between October 2018 and July 2019, reaching an overall coverage of almost 100% throughout this period. Activities to support the response were instituted, such as advocacy missions, AFP monitoring, environmental surveillance, and surveillance strengthening through the use of AVADAR, eSurv, ODK, ISS, and SIAs. This was an opportunity to use these tools to validate data from the field. General census staff also were used and the border regions were covered. The information showed how the vaccine was used as opposed to how it should have been used, and through GIS, it was established that some households had not been reached even with the 100% coverage reported and thus some of the districts were disqualified. The data from the field now is of good quality. Response coordination required the commitment of politico-administrative and customary authorities at all levels, deployment of an incident manager, daily monitoring of preparations and implementation of vaccination activities, meetings with officials of border districts in Nigeria and Chad, and evaluation meetings on the status of implementation of immunisation activities at district, regional, and national levels.

The main challenges to the response efforts were: (1) maintaining a high level of commitment among the country’s authorities at all levels; (2) involvement of other ministries and actors besides the Health Ministry, such as the defence and security, interior, communication, education, hydraulics, and livestock ministries; (3) sustaining cross-border collaboration with Nigeria and Chad; (4) reaching children in special populations such as nomads, refugees, and displaced persons; (5) reaching nomadic populations in seasonal transhumance between Nigeria and Niger; (6) vaccinating children in areas bordering Nigeria and in the islands of the Lake Chad Basin where there is insecurity from armed groups and banditry; (6) vaccinating urban and peri-urban populations; and (7) insecurity resurgence mainly along the Niger–Nigeria borders from attacks by armed groups such as Boko Haram, and in Tesker, Goudoumaria, Mainé-Soroa, and N’Gourti, as well as the increase in kidnapping incidents since the beginning of 2019.

### Implementation of geographic information systems and innovative technologies in Nigeria: policies, applications, challenges, and lessons learnt

#### Daniel Oyaole

##### World Health Organization Nigeria Country Office, Abuja, Nigeria

In terms of GIS readiness, Nigeria’s National Primary Health Care Development Agency has no certified GIS capacity or human resources. Support of GIS at the Emergency Operations Centre is provided by WHO and BMGF. The use of GIS in public health has been in satellite imagery, SIAs, surveillance on achievement of AFP core indicators, locating cVDPV2 and affected/susceptible regions, electronic active surveillance, improvement of access to data, and building capacity through the Geo-Referenced Infrastructure and Demographic Data for Development (GRID3) programme. This programme is part of a global initiative that aims to improve access to data for decision-making in participating countries. It collects geospatially referenced data relevant to a variety of sectors, which are then housed in the GRID3 portal and are open to all stakeholders.

Satellite imagery has been used to map inhabited and uninhabited areas, changes in population movement, and vaccination and surveillance reach. In Borno State, for example, 84% of 22,556 geolocations were reached by surveillance activities and the use of updated satellite imagery led to a significant drop in the number of children unreached with vaccination, eg, going from 102,000 in May 2018 to 71,000 in October 2018. This trend has continued and the level was at 43,507 in June 2019. In regard to SIAs, GIS tools serve to track vaccinations, determine geographic coverage by settlement and administrative level, and generate related data. The data are Web based and are accessible to all levels for corrective action. Surveillance activities involving GIS tools check the achievement of AFP surveillance core indicators, and monitor, through eSurv, the activities of government personnel and visits to priority sites and health facilities. These tools help in locating cVDPV2 and affected/susceptible regions, which is part of the polio-risk analysis that is used to determine outbreak-response scope.

Challenges to GIS implementation include: (1) incomplete ward-level maps, and standardisation of names and codes at the health-facility level; (2) limited GIS capacity and constrainment of training opportunities by the conditions of the ArcGIS license; (3) nonexistence of policies supporting GIS and information sharing; and (4) the effect of competing priorities and workloads on achievement of goals.

Lessons learnt include: (1) GIS activities were instrumental in ensuring the quality of the polio campaign; (2) through tracking of vaccination teams, VTS ensured that all areas were covered; (3) GIS mapping provides good insight to policy makers through spatial data analysis and gap identification; and (4) geodata gives WHO PEP a better understanding of the areas of its operation and their populations.

### Mapping Somalia

#### Frank Salet

##### Bill & Melinda Gates Foundation, Seattle, Washington, USA

A large amount of data has been collected over the last few years in Somalia, mostly using the ODK phone application. Many of the data points have GPS coordinates, which when compared with the population density grid show the locations of polio programme activities and where they are absent. All the internal geographic boundaries have been redrawn based on the needs of the polio programme and show inaccessible areas. Locations of cVDPV2 and cVDPV2 cases have also been identified. All the positive cVDPV2 environmental samples were isolated from the Banaadir region. Somalia does not have a master health-facility list, but an operational version with names and codes of 800 health facilities was produced by the polio team. Surveys were also conducted to assess cold-chain availability and national Expanded Programme on Immunization service capacity. Somalia has ~ 400 vaccination posts, more than half of which are located at key passage points between accessible and inaccessible areas or at international boundaries.

Approximately 11,000 settlements were mapped. To understand the population dynamics for better targeting of different groups, the polio programme classified each settlement based on the four population groups and accessibility. All analysis of activities was segregated based on this classification. For nomadic groups, a list with contact details of 1302 elders was compiled by the United Nations Children’s Fund. Somalia has 2.6 million internally displaced persons (IDPs) in 1893 IDP sites. Besides WHO staff, community health workers and village polio volunteers are involved in ongoing surveillance, particularly in inaccessible areas.

Since 2016, all operational data have been entered and collected using the ODK electronic system. Reference maps and polio overview maps were produced for each province and region, with district boundaries, health facilities, settlements, roads, rivers, IDP camps, and population. Detailed maps for microplanning were produced for all of Somaliland, Puntland, and Banaadir. Various mapping sessions and microplanning workshops were held, and large maps were produced, printed, and distributed. Supplementary immunisation activity monitoring was tracked through ODK, showing each household visited and households with children not finger marked.

### Country experience in implementation of electronic surveillance: Uganda

#### Paul Mbaka

##### World Health Organization Uganda Country Office, Kampala, Uganda

In June 2019, Uganda detected an EVD case in Kasese and immediately put in motion rapid-response mechanisms for disease surveillance and management. ODK is used for community-based surveillance data collection. These data are presented in many ways, including in charts showing alerts from the community. The reporting of suspected events uses an SMS-based system that is integrated with DHIS2 and the mailing system, and details of any cases are entered into DHIS2. ODK is used for analysing the information.

ODK-based surveillance is used during EVD detection. It provides details on the locations visited for viral haemorrhagic fever surveillance. The DHIS2 system is used for reporting and case registration, while during investigation, an SMS-based system sends out messages for sample tracking. Laboratory results are entered in DHIS2 for immediate access. Other investigation tools at the community level are based on electronic surveillance, including AFP case investigation using ODK, which is meant to provide the national task force with additional information on the situation on the ground. This information is presented through the EVD dashboard to show the cases, districts affected, and financial and surveillance information.

Ebola virus disease response involves use of an integrated eSurv system that encompasses several tools: (1) Go.Data is used for monitoring viral haemorrhagic fever events, cases, and their contacts, which are then used to generate charts and risk-approximation data, and for case-management training; (2) ODK is used in pre-, intra-, and post-measles-rubella-polio campaign monitoring and supervision, and case geocoding and verification; (3) DHIS2 is used in monitoring disease trends; and (4) the SMS-based mtrack is used in disease surveillance.

Current developments and future plans include: (1) introducing social media monitoring for epidemic intelligence gathering from open sources, speech-to-text innovations, etc., as a way to generate data that managers and policy makers can use, since they find products from tools such as eSurv hard to understand and use; (2) forecasting using predictive analytics and population-movement mapping using mobile-phone data; (3) updating the master facility register and harmonising GIS coordinates; (4) integrating dashboards using DHIS2 and ODK data in a single platform to provide managers with a comprehensive view of the process to earn management-decision support; and (5) training additional users.

### Why the Bill & Melinda Gates Foundation cares about geographic information systems

#### Vince Seaman

##### Bill & Melinda Gates Foundation, Seattle, Washington, USA

The BMGF strives to accomplish its mission of ensuring that ‘every person deserves the chance to live a healthy, productive life’ by improving the quality of life of the world’s poorest populations. To help people in developing countries, however, we need to know who, where, and how many they are. Until recently, BMGF relied on administrative data reported at administration levels 1 and 2, which have serious limitations: the quality cannot be validated or verified; they are collected at preset intervals, and thus it is difficult to measure change and impact; and they cannot target subgroups or vulnerable populations. Geographic information system data can point out specific areas in need of food, medicine, vaccines, and other life-saving interventions, as well as how many people are affected. Equally important, GIS data allow better measurement of the quality and impact of interventions. The first BMGF investment to focus on GIS innovations was support for polio eradication in Nigeria and it was the genesis of numerous projects in a variety of programme areas that used GIS tools like those developed for polio. Nigeria was chosen because in 2012, it was found out that polio cases were on the rise in northern Nigeria, and one suspected cause was the use of incomplete and inaccurate microplan maps.

The BMGF supported the mapping of 10 northern states where polio was endemic and found that ~ 5% of settlements seen on satellite imagery were not part of existing polio microplans. The GIS maps were used to support microplanning and as a reference layer for vaccination team tracking. The main value of the tracking was to identify settlements that had not been visited. These efforts led to elimination of polio in accessible areas of Nigeria by mid-2014. Accurate ward boundary maps allowed creation of fixed-post vaccination campaign maps that ensured no child would need to travel > 1 km to a vaccination post.

The operational local government area (LGA) boundaries created by aggregating ward data did not align with existing boundary shapefiles used by the government of Nigeria and the United Nations. This would affect spatial data such as the location of a polio or measles case, which could be allocated to the wrong LGA. Population estimates using raster GIS data also can be affected by inaccurate boundaries. A detailed investigation using census enumeration area maps determined that the government of Nigeria and United Nations boundary shapefiles were incorrect, and the VTS boundaries more accurately reflected where the census was conducted.

Outputs and impacts of this project include: (1) a nearly completed basemap for Nigeria that contains all major settlement names and locations, key points of interest, and boundaries that are more accurate than existing government and United Nations data sets; (2) unique, bottom-up population estimates produced as a 100-m gridded raster layer; and (3) other BMGF programmes now introducing many of the GIS innovations in their strategies, including for malaria, neglected tropical diseases, vaccine delivery, nutrition, family planning, drinking water, sanitation, and hygiene. This work has a large footprint in Africa and Asia, including in Afghanistan, Cambodia, Cameroon, Democratic Republic of the Congo, Pakistan, and Somalia, as well as Haiti. In all of these countries, there were no spatially accurate maps or boundary shapefiles at the national level, similar to the situation in Nigeria.

Only country governments can effectively collect their own reference data and authorize their use, but most lack the necessary technical capacity and resources. To help solve this problem, BMGF is: (1) partnering with the UK Department for International Development on a GRID3 project that is working with federal and state government teams to complete the mapping in Nigeria, and to build the local capacity to accurately collect and validate spatial data (note: GRID3 is replicating these efforts in five other countries and is expected to continue to expand thru 2023); (2) supporting the AFRO GIS Centre, which strives to develop GIS and data-management capacity in the WHO African Region, including helping governments validate and improve their existing boundary shapefiles; (3) supporting the MapAfrica project, which provides the Ecopia AI (Ecopia Tech Corporation, Toronto, Ontario, Canada) building footprints and roads for all countries in sub-Saharan Africa, with the underlying imagery available for validation purposes (note: the footprint layers will facilitate accurate mapping and population modelling, and validation of existing spatial data sets); and (4) making all spatial data collected under BMGF investments that do not contain private personal information accessible on the GeoPoDe Website.

There remains a need for alignment and coordination in the global community to provide the support and technical guidance needed to produce accurate spatial reference data, and create globally accepted data-quality standards and best practices. It is hoped that the AFRO GIS Centre, and by extension the GIS focal points in the Member States, will help overcome these barriers and lead the region into a new GIS era, supporting not only polio, but all regional activities.

### Geographic information system bottom-up population estimates: Volkswagen to Cadillac options

#### Vince Seaman

##### Bill & Melinda Gates Foundation, Seattle, Washington, USA

During 2013–4, the time when GIS mapping was taking place in northern Nigeria, it was noticed that administrative population denominators were often much higher than census projections or estimates based on viewing settlements using high-resolution satellite imagery. Because the census data were > 10 years old and of questionable quality, the polio programme needed a better source of denominator data and one that was independent from census or administrative data. Utilizing imagery and settlement maps developed for polio microplanning and tracking, along with targeted microcensus work in selected states, made it possible to develop the first bottom-up modelled population estimates at a national scale. The output is a 100-m gridded raster layer that can provide total population or a selected demographic. The data can be downloaded from VTS and the GeoPode Website, which also have a number of user-friendly tools to query population for custom areas.

Lessons learnt included: (1) Census projections are not reliable: a yearly growth rate of 2.7–3.4% is applied at the state level to create census projections, but states have highly varying growth rates, ranging from 2 to 6%. At the LGA level, the variation is even wider, ranging from 1 to 10%. For slow-growing states, 10 years of flat-rate growth will generate census projections that are too high and the opposite is true for fast-growing states. (2) Fixed demographic fractions are not representative: Nigeria uses fixed fractions for key target populations, ie, U1 = 4%, U5 = 20%, and U15 = 46.7%, but DHIS2 survey data modelled across the country show that the fractions vary widely, eg, U5 ranges from 11% in the south to 23% in the north. (3) Inaccurate boundaries result in inaccurate population estimates: the official government of Nigeria and United Nations boundary shapefiles for Nigeria were found to be inaccurate during the mapping exercise. If they are used to query population from a raster, the results will not be accurate.

Most countries will not have the resources to create the Cadillac version of the bottom-up Nigeria model; however, with the imminent availability of the building footprint layer for all of sub-Saharan Africa, a much less expensive process is possible. The AFRO GIS Centre, with the support of partners, will endeavour to create a basic population layer for the region using the building footprints and available survey data. If desired, individual countries can then improve these estimates by collecting additional microcensus data if and when they have the resources.

### Foundation of using Tableau for visualisation

#### Nick Hara

##### Tableau Foundation, Seattle, Washington, USA

The Tableau Foundation works to accelerate the use of data in solving the world’s problems through helping people see and understand data. Using smart analytics, the foundation is fostering collaboration, making it easier to share data and develop holistic solutions to problems. In public health, part of tackling disease is understanding the scope of the issue, ie, where it is concentrated, who is affected, and what resources exist and how people can be connected to them. That information can be provided through data analysis. The foundation partners with organisations on the frontlines tackling issues of global health, and equips them with the software to enable comprehensive data collection and analysis for the greatest impact.

People working with country health institutions need to present health data in formats that will allow decision-making officials to understand the situation and provide their support for proposed interventions, which will also make it possible for health workers at every level to get ahead of a disease and deploy resources accordingly. The Tableau Foundation facilitates that by providing a platform that fosters practical applications of data.

## Side event presentation summaries

Two side events were organized to steer participants to review, reflect, and provide input on the integrated data system for managing polio data and pathways to developing national strategies for adoption of GIS in Africa. The specific objectives were:

- To review and discuss the WHO Polio Information System (POLIS), its data use, and current status; and
- To review and discuss the global strategy on digital health (2020–4) and how it is being leveraged for national strategies for adoption of innovative technologies and GIS in Africa.

### The WHO Polio Information System

#### Ashely Burman^1^ and Ravi Santhana^1^

^*1*^ *World Health Organization, Geneva, Switzerland*

The POLIS is an integrated approach to harmonize all polio resources for effective resource utilization. Undoubtedly, the innovative and technologic tools and resources, developed and deployed under the polio program was the game changer in the war against polio in the last 3 decades. These innovative tools were the offshoot of the RCC in Douala in 2017.

**Fig. 1:**
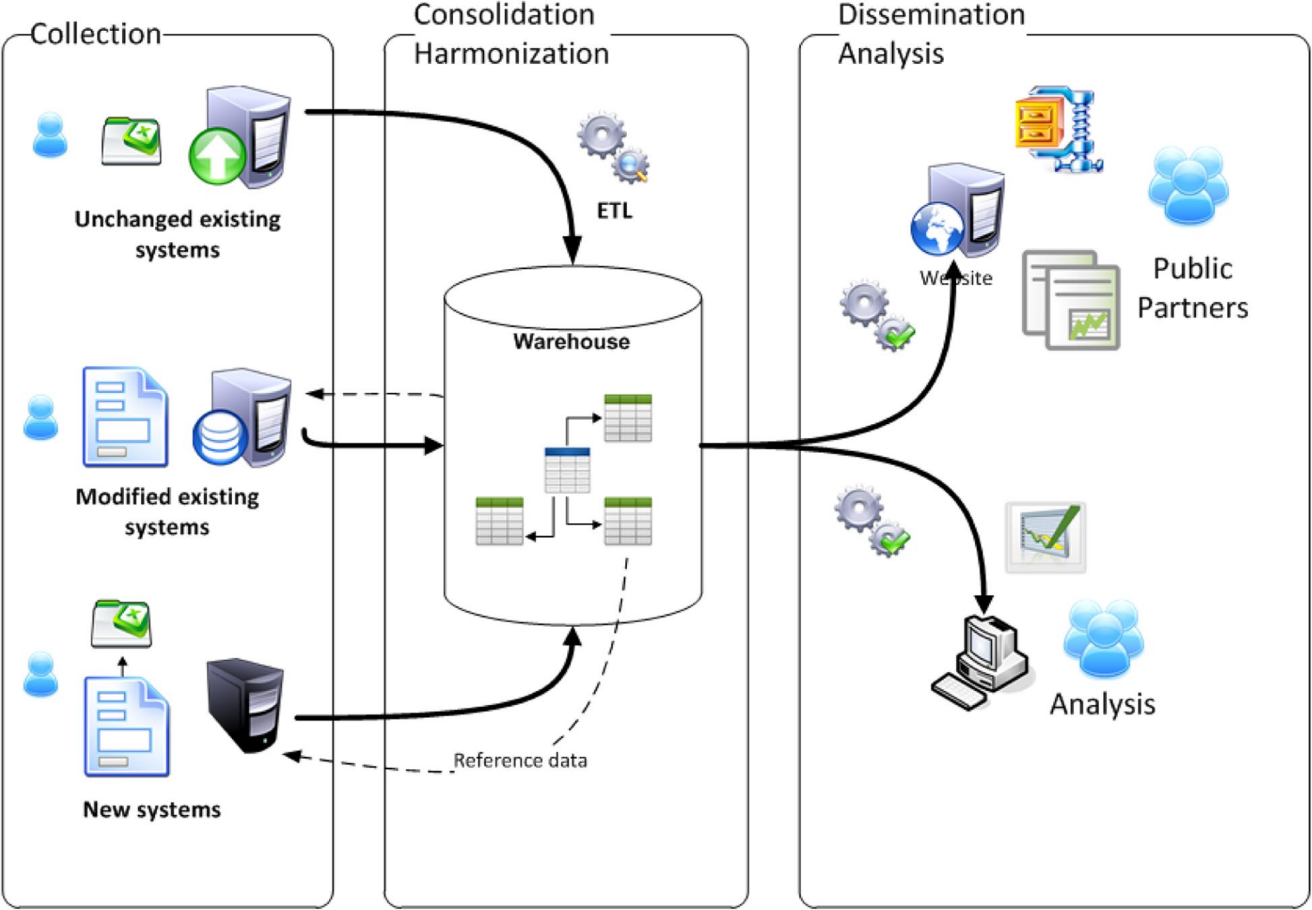
POLIS workflow: An integrated approach to polio data management. ETL, extraction, transformation, and load

The POLIS looks at consolidating these tools in three approaches:

- *Data collection:* Laboratory, AFP case based, specimen samples, environmental surveillance samples, and immunization data.
- *Data harmonization:* Interoperable interfaces.
- *Data dissemination*: Data visualization tools, dashboards, reports, line lists, journals, peer-reviewed papers, publications, advisory notes, etc. (Fig. 1).

##### User group and data sharing

Each core GPEI partner is authorized by the WHO to share AFP and WHO geodata for the purpose of analyses, mathematical modeling, and risk assessments with a collaborating institution (“collaborator”), which has been approved by the WHO and has agreed to be bound by the same terms and conditions as those under which the data were provided to the core GPEI partner. In February 2013, GPEI finalized this data sharing agreement with all partners and governments that have POLIS as a warehouse for polio data sets; the shared data sets include AFP, laboratory, environmental surveillance, SIA (including inside house monitoring and lot quality assurance sampling), and geo-data.

##### Components of the WHO Polio Information System

The components of POLIS include a login page, dashboards, outbreak response indicators, a search option, reports, community of Practice, and contact details.

##### How the WHO Polio Information System works

The POLIS receives input from the Global Polio Laboratory Network (laboratory test results), POLCASE (polio case management application; AFP and environmental sample data), POLSIA (immunization activity application; immunization, vaccination, and monitoring data), and POLGIS (population and geographical reference data), as well as POLADMIN (system administration application), and provides outputs in the form of reports, dashboards, maps and line lists. The outputs can also be exported or linked to another system with the use of appropriate APIs and credentials.

The POLIS affords access to a community of practice where related issues and topics are deliberated.

### National strategy for adoption of innovative technologies and geographic information systems

#### Derrick Muneene^1^ and Hani Farouk^2^

^*1*^ *World Health Organization, Geneva, Switzerland.*

^*2*^ *World Health Organization, Regional Office for Africa, Brazzaville, Congo*

##### The World Health Organization Triple Billion agenda

In closing the 71st World Health Assembly in May 2018 in Geneva, Switzerland, the WHO Director General said that everything the WHO did going forward would be evaluated in the light of the “Triple Billion” targets, which were approved in the WHO’s then-new 5-year strategic plan. By 2023, the Triple Billion targets aim to achieve:

- 1 billion more people benefitting from universal health coverage;
- 1 billion more people better protected from health emergencies; and
- 1 billion more people enjoying better health and wellbeing.

The Digital Health unit is located within the Health Systems and Services Cluster in the Health Information and Knowledge Management unit. The Digital Health unit supports the 47 member states in the following key areas:

- Digital health strategies and regulations;
- Digital health capacity building; and
- Digital health solutions (addressing the growing need for paperless patient-centric solutions that cover key health events).

The Digital Health unit also supports intercluster support on digital health, including GIS. Health system strengthening in the Sustainable Development Goals era requires considerable investment in digital health. The World Health Assembly, through resolution WHA58.28, urged member states to take affirmative action in introducing ICT to the health sector and providing an enabling environment for its usage as eHealth, as well as establishing eHealth strategic plans, ICT infrastructure, data standards, public private partnerships, national centers of excellence, and national health information systems. Resolution WHA66.24 66.24 further stresses the need for eHealth interoperability and data standards to ensure seamless eHealth operations and services, data security, and reduction of fragmented eHealth solutions.

The WHO AFRO, in resolution AFR/RC60/R3, passed actions on eHealth solutions in the African region, emphasizing the needs for political commitment, formation of eHealth champions, eHealth governance mechanisms, multisectoral coordination, ICT infrastructure support, ICT curriculum in health learning institutions, and monitoring and evaluation of eHealth.

Resolution WHA 60.29 on health technologies affirms that medical devices are indispensable tools in the process of providing care to patients, and in delivering preventive, diagnostic, treatment, and rehabilitative health services. Resolution WHA 71.7 further stresses the need to harness digital health, digital health capacity building, intersectoriality, and donor coordination.

**Fig. 2:**
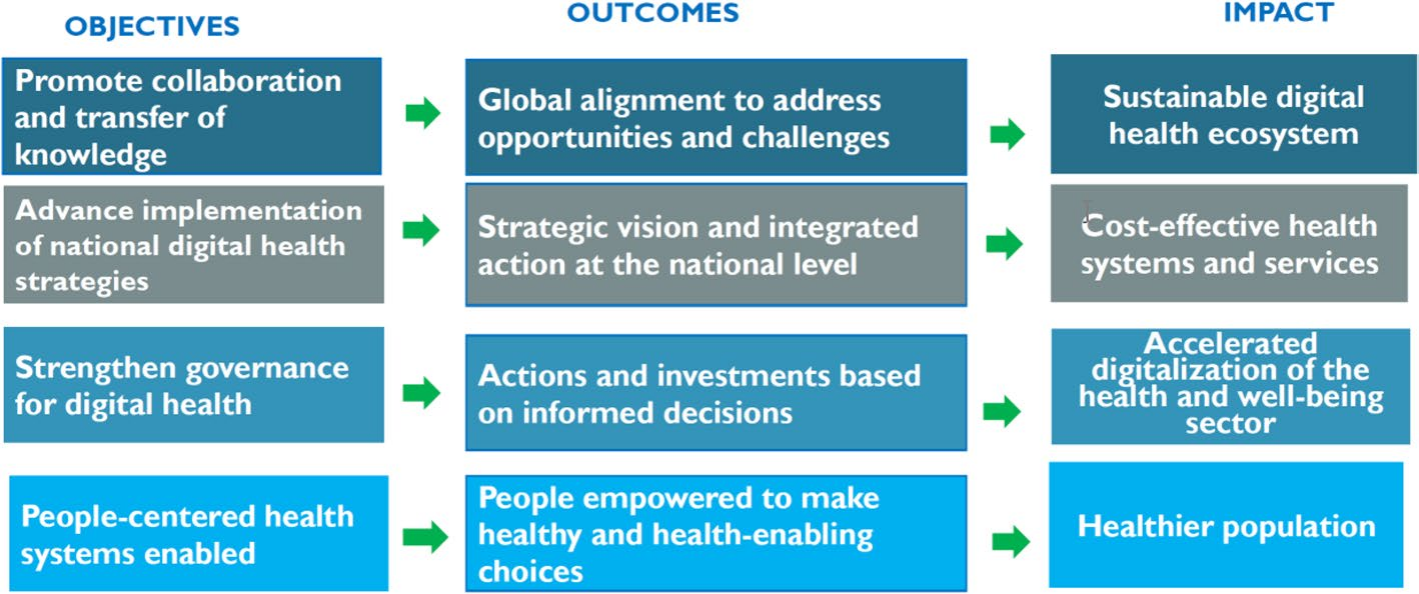
Action plan: Global strategy on digital health 2020–4

The vision of the global strategy is to improve health for everyone, everywhere by accelerating the development and adoption of appropriate digital health solutions to achieve the health-related Sustainable Development Goals through four strategic objectives:

1. Promote collaboration and advance the transfer of knowledge on digital health;
2. Advance implementation of national digital health strategies;
3. Strengthen governance for digital health at global and national levels; and
4. Advocate for people-centered health systems enabled by digital health (Fig. 2)

Digitization challenges include limited token/involvement of users, pilot-focused initiatives, closed systems, vertical silos systems focused on disease/business processes, and security and privacy/identification issues. Other key GIS issues include GIS in ICT strategies, training on GIS, hardware (GIS server) at country level, software (open source/licensed), funding, and project management.

## Discussion

Discussions at the meeting generated a wide variety of questions and observations that could be of value to WHO Member States in their efforts to adopt GIS innovations in public-health service delivery.

### Data and technology

One size does not fit all. The ability to use data-intelligence tools depends on country capability and resources, and if reference data are available from other sources like WHO, they could serve as a foundation. The choice of application should be defined by whether it is providing value for money and doing what needs to be achieved. Countries should look at the resources they have and how many variables need to be captured. They need to prioritize their needs, and can start small and expand as needed. An important consideration is capacity of the software for integration with other systems. Interoperability of systems is essential and unless data systems are talking, problems will result.

Software capability and data quality should not be confused. Software will not fix bad data quality. Geographic information systems and other information tools are just one component of the information system. They facilitate collection, processing, and communication of information, but the quality of their product is determined by the quality of information input. In public-health service supervision, GIS software will indicate a physical visit was made, but not the nature or quality of the work performed. Other methods can be used to verify the nature and quality of the work, such as taking photos during site visits and checking vaccination cards. The WHO Member States need to come up with efficient processes of ensuring that the value and intended use of information they collect are communicated during planning for data collection, and that the data collected satisfy that purpose.

Many countries have data on their health facilities that would be useful for public-health purposes, but are scattered in various locations, are not shared, and in many cases, are not validated. Such data should be integrated for access by relevant actors. National governments and subnational teams have to be involved to ensure that the AFRO GIS Centre has access to such data for its work and for their appending to the global database. The validation process applies to the administration boundary data set, names of locations, and topology. Spatial data need to include specific place names to be of value and these can be collected over time. A master facility list is also essential.

Availability of data through software tools does not equal free access. Permissions will be required for sensitive data. Countries should have a policy and a process to facilitate data accessibility, while ensuring that sensitive data that are important for security reasons are secured.

Planning for and collecting GIS data before an outbreak are very important for resource mobilisation and resource allocation. Searching for missing information during an outbreak is time wasting and could contribute to mortality levels. There is also a need to distinguish between regular and emergency GIS work, where there is a need to move quickly to feed the dashboard with information.

Although GIS and other software tools save lives in public-health service delivery, they often are met with resistance mostly associated with the perception that they require extra effort to learn and use, and are costly. Advocacy and education on these tools will be required on their time-saving, efficiency, and empowerment benefits.

### Workforce

Normally, training on innovations is introduced to technicians first, who then are required to sell the innovation to the relevant decision makers. To bring policy makers on board for resource provision, it is necessary to demystify technology so it is understandable for them. It may also be necessary to change the approach to training to start with the decision makers, eg, ministers and directors, so that when technicians are being trained, the technologies have already gained acceptance at higher levels.

Online training is not adapted for policy makers, and GIS courses targeted to that group would facilitate their involvement in and support for GIS. The WHO could gather such resources and make them available to the Member States for access by policy makers.

The focal points have the responsibility of spreading information about GIS and other innovations to all the health resources in their countries to ensure they have access to the relevant information available from the WHO Regional Office for Africa to run advocacy campaigns.

The WHO Regional Office should work to develop a mentorship programme within the African Region to contribute to long-term capacity building. In addition, it will need to consider helping countries and their respective ministries of health to acquire the capacity to manage reference data, which is the backbone for materialisation of the GIS vision.

### Use of geographic information systems outside polio

Geographic information systems can be applied in programmes other than polio, eg, neglected tropical diseases and routine immunisation. For EVD in the Democratic Republic of the Congo, GIS use with support from satellite imagery allowed identification of locations in the equatorial forest that had not been reached with surveillance, facilitating direction and focus of actions to contain the disease. The direction the WHO Regional Office is taking is to foster GIS use across programmes and issues related to the application of GIS are discussed monthly at the director level. Uptake has not been high, but there is a major commitment by the WHO Regional Office. In addition, some programmes have started using GIS, including for immunisation. The countries also, however, need to take the initiative and find innovative ways to use GIS in other programmes, based on country needs and capacity. It is up to the countries to go beyond what is proposed from the regional program to be innovative in GIS application.

### Governance

Recognition of the issue of political interference is important in boundary definition because political leaders, particularly in conflict-affected areas, would want to control these definitions for political purposes. Boundary definition, whether for settlements or health-catchment areas, can cause conflict, but such conflict can be avoided if leaders are brought on board early in the process and when the boundaries are labelled in nonpolitical terms, such as operation or vaccination boundaries. Government boundaries would not be appropriate for health purposes because they are based more on terrestrial circumstances rather than population structures. To compound the problem, most countries have multiple agencies dealing with boundaries.

Identifying the GIS entry point can be difficult for a country, and establishing guidelines for application across countries cannot work because needs, resources, and capacity vary. Normally, innovation comes before regulation. An innovation is presented to a government and then given a home in a policy document. It is important that structures be in place for innovations to ensure that they are sufficiently regulated. Development-partner conditions can interfere with a country’s priorities and governance for innovations. Legislation and policies need to provide for regulation of development-partner interventions, and to require that such partners work with relevant stakeholders and submit the outputs from their activities to relevant institutions. In many cases, partners bring their innovations into a country during emergencies when that capacity is missing and thus there is a need to cater for such situations in legislation.

For harmonisation, countries should consider having one eHealth strategy in which GIS is included. Some countries have two governance structures, with ICT at the ministry level and a technical working group. Interministerial collaboration is necessary for all components of health decision-making to be harmonised. Although the ministries of health and ICT may work well together, there is a need to go beyond ICT to include other relevant ministries, such as security, which should be involved from the beginning to ease the path to adoption. The GIS strategy does not have to be a unique document, but can be integrated in the policies, strategies, and action plans of relevant ministries. Most countries will not have to start from scratch, but will just need to tweak their strategies to accommodate or give priority to the new technologies.

## Next steps

Planned short-term follow-up action after the meeting will include production and sharing of the meeting report and all technical documents, communication and teleconferencing on meeting recommendations and country-level feedback, and resource mobilisation to support planned activities. The countries will be expected to come back to WHO with specific requests for capacity building for their planned projects. In the medium term, ie, over the next 1–2 years, the AFRO GIS Centre will work with the countries and the support of different stakeholders to implement the GIS road map, and organize a review and planning meeting to follow up with different stakeholders on progress.

### Immediate action points

- Given that all countries have focal points, WHO will ensure that they have access to all the information they need and open an account for them in the framework of GIS.
- Completing and updating the lists of focal points by the countries are important for facilitating coordination and planning activities.
- The WHO has many templates for activity-based or genetic geographic data profiles. Country representatives should connect with the AFRO GIS Centre to obtain the templates and profiles for their country as a base to build on. The WHO will share information on codes and standards for the available countries’ geographic profiles.
- Countries will need to collect data sets to feed into regional and global geodatabases. The AFRO GIS Centre will work with the WHO Reference Centre to develop 1-page documents to provide guidance so that data the countries collect will be in a similar format for ease of sharing.
- The AFRO GIS Centre will send meeting participants a 1-page document on how to collect geocoordinates, as well as share the Point of Interest and Knowledge (POINK) application to support data-collection activities, which can also be used to determine if the countries need more support.
- All meeting participants will be added to the WHO African Region’s GIS network and its listserv (AFGISNET@listserv.who.int) to allow communication and sharing of data and knowledge.
- The WHO will provide technical support to Member States to establish their national enterprise GIS server for security assurance.

### Polio Eradication Programme research grant

To jumpstart or enhance the adoption of GIS and related technologic innovations for health decision-making in the WHO Member States, PEP has introduced a grant that will provide seed money support for research demonstrating innovative use of GIS and other technologies for polio eradication, and the documentation, validation, and promotion of GIS technology for public health across the WHO African Region. Such technologies and innovations will include: GIS, remote sensing, modelling, spatial, and geostatistical analysis; mobile technology use for field-data collection; population-estimation techniques and crowdsourcing for data collection and validation; artificial intelligence and machine learning; use of drones for vaccine delivery; business intelligence and information use; and data management, and integration and automation of data flow and big data.

Details on the grant and a copy of the letter of interest template are available for the African Region at: https://www.afro.who.int/health-topics/polio/research-grants-gis-polio-eradication.

## Recommendations

Two sets of recommendations were produced at the meeting: general recommendations applying to WHO Member States and WHO without regard to Member States’ GIS capacity, and specific recommendations defining actions for Member States, WHO, and partners based on Member States’ GIS capacity. An analysis of the recommendations was performed to develop a base for the WHO African Region’s national GIS road map, which was intended to: (1) provide an overview of the status of the three GIS pillars in each country as a starting point for determining the nature of support a country would require to implement GIS for health decision-making; (2) form the foundation for a GIS road map for each country, indicating priority activities to be performed with the leadership of the respective ministry of health; and (3) serve as a general monitoring tool to track Member States’ progress towards implementation of sustainable GIS services and applications for health decision-making. The road map was developed in MS Excel to allow the countries to: (1) document and estimate their level of adoption and implementation of GIS; (2) identify, prioritize, and detail their activities in a national plan for the period 2020–1, indicating responsible teams, duration, monitoring aspects, and cost; and (3) provide a summary of progress on implementation, and documentation issues, challenges, and lessons learnt.

### General recommendations

#### World Health Organization Member States

- Develop strong governance mechanisms and bring all stakeholders on board, including nontraditional actors such as the respective security ministries, particularly in the constitution of the steering committee, while being strategic about representatives to be included, and their roles in the adoption and use of the innovations.
- Facilitate intersectoral collaboration and ensure communication channels are open, especially where multiple agencies deal with boundary definition. Undertake advocacy and capacity building, and facilitate interministerial collaboration in the development of a comprehensive and all-inclusive digital-health strategy.
- Determine entry points for GIS systems. It is important that structures be in place for GIS innovations and the ministries must bring this to fruition through the interministerial collaboration structure. The GIS strategy needs to be integrated into the countries’ policies, strategies, and action plans.
- Oversee and coordinate involvement of partners, and ensure outputs from their activities are accessible to relevant government agencies and other stakeholders who would benefit from them. Create a policy requiring partners to involve country stakeholders in data-collection activities.
- Conduct a health-system stakeholders’ awareness forum on the use of ICT to generate greater buy-in for implementation of plans resulting from the strategies adopted.
- As part of emergency preparedness, put in place systems that facilitate the use of GIS during emergencies for speedy delivery of interventions. For example, ensure that master lists of health facilities and population settlements are developed and updated regularly, and maps with updated subnational boundaries are available, and determine the definitions and levels of health facilities, and treatment and service-delivery sites, which are essential to supporting interoperability, information sharing, and decentralised data management.

#### World Health Organization

- Support capacity building for Member States in technical and technologic domains, such as GIS, data management, business intelligence, and information visualisation, so they can do the work themselves.
- Work with Member States to develop a geocoordination working group tasked with setting up a mentorship programme.
- Provide guidance on GIS tools, including on their functionality, licensing, and sustainability.
- Develop a document to serve as a guide for the countries in their choice of data-analysis tools and methodologies.
- Provide guidance to Member States on data accessibility, helping define what should be considered sensitive data of importance for country security, whose access will be restricted, and what can be freely accessible.
- Emphasise the building of self-financing capacity and innovative financing to support development of strategies and exploitability of solutions to be implemented for the entire health system.
- Establish mechanisms for countries to benefit from the experience of Nigeria and the WHO Regional Office for Africa in GIS and innovative technologies.
- Develop a platform for Member States and partners to provide: (1) an online repository to document and share experiences, success stories, and details of national projects in the domain of GIS and innovative technologies; (2) a workspace for data collection, validation, and sharing; and (3) a repository of minimal sets of standards.
- Identify capacity-building opportunities in collaboration with partners for the benefit of Member States.
- Assemble a group of governments and academic institutions to negotiate and draw up a memorandum of understanding for access to updated satellite imagery such as DigitalGlobe (Maxar Technologies, Westminster, Colorado, USA).
- Support knowledge transfer for academic writing and research methodology development via a series of Webinars, with a focus on spatial analysis and process.
- Promote the use of free and open-source tools that can be deployed at the national level to support GIS applications and services of the respective ministries of health.
- Promote the adoption of systems, data structures, and tools developed for polio eradication, eg, field-data collection tools, tracking systems, spatial analysis, standard operating procedures, templates, and guidelines, and share source code/software applications, and available data sets and structures.
- Provide technical support on data management and information use.
- Provide technical support for the development of national strategies, plans, fundraising proposals, and concept notes for GIS implementation.

## Data Availability

Not applicable.

## Abbreviations

AFP: Acute flaccid paralysis
AVADAR: Autovisual AFP Detection and Reporting
BMGF: Bill & Melinda Gates Foundation
cVDPV2: Circulating vaccine-derived poliovirus 2
DEMs: Digital elevation models
DHIS2: District Health Information Software 2
ESPEN: Expanded Special Project for Elimination of Neglected Tropical Diseases
eSurv: Electronic surveillance
EVD: Ebola virus disease
GIS: Geographic information system
GPEI: Global Polio Eradication Initiative
GPS: Global Positioning System
GRID3: Geo-Referenced Infrastructure and Demographic Data for Development
ICT: Information and communications technology
IDPs: Internally displaced persons
ISS: Integrated supportive supervision
LGA: Local government area
NTD: Neglected Tropical Disease Programme
PEP: Polio Eradication Programme
POLIS: WHO Polio Information System
RRT: Rapid Response Team
SIAs: Supplementary immunisation activities
SMS: Short message service
VPDs: Vaccine-preventable diseases
VTS: Vaccination Tracking System
WHE: World Health Organization (WHO) Health Emergencies Programme
WPV: Wild poliovirus

